# Anatomical Evaluation of the Pterygomaxillary Complex Using Cone Beam Computed Tomography

**DOI:** 10.3390/tomography12010009

**Published:** 2026-01-09

**Authors:** Ömer Demir, Kamil Serkan Ağaçayak

**Affiliations:** Department of Oral and Maxillofacial Surgery, Faculty of Dentistry, Dicle University, 21280 Diyarbakir, Türkiye; serkanagacayak@gmail.com

**Keywords:** Le Fort I osteotomy, pterygomaxillary complex, CBCT, morphometry, surgical anatomy, complication prevention

## Abstract

Background: The pterygomaxillary region is a complex anatomical area formed by the junction of the maxillary, palatine, and sphenoid bones and contains critical neurovascular structures. Accurate assessment of this region during Le Fort I osteotomy is essential, particularly to prevent hemorrhage and nerve injury that may occur during the pterygomaxillary separation phase. This study aims to investigate the morphometric characteristics of the pterygomaxillary region using cone-beam computed tomography (CBCT) and to evaluate the effects of age, sex, and laterality on these anatomical parameters. Materials and Methods: In this retrospective study, CBCT scans of 200 individuals (100 males and 100 females) aged 20–80 years were analyzed. Axial measurements included distances between the piriform rim, the descending palatine artery, the pterygomaxillary osteotomy line, and the pterygomaxillary fissure. Additionally, the thickness and width of the pterygomaxillary region and pterygoid process, lengths of the medial and lateral pterygoid laminae, and the distance between the greater palatine canal and the medial pterygoid lamina apex were recorded. Measurements were statistically evaluated by sex, age group, and laterality. Results: The following parameters demonstrated statistically significant differences based on the conducted measurements: The distance between the piriform rim and the descending palatine artery was significantly greater on the left side (*p* < 0.001). The length of the lateral pterygoid lamina increased with advancing age (*p* = 0.048). The thickness of the pterygomaxillary region was significantly greater in females (*p* = 0.014). Additionally, the distance between the greater palatine canal and the terminal point of the medial pterygoid lamina was significantly higher in males (*p* < 0.001). Conclusions: The pterygomaxillary region exhibits anatomical variations that may lead to serious complications during Le Fort I osteotomy. Detailed preoperative evaluation of this area using CBCT can guide surgical planning and help prevent potential vascular and neural complications.

## Simple Summary

The pterygomaxillary region is a sensitive anatomical area that contains important vessels and nerves and may be at risk during Le Fort I surgery. In this study, cone-beam computed tomography (CBCT) images were used to evaluate how the shape, thickness, and width of this region differ according to age, sex, and side of the face. The results showed clear anatomical variations between individuals. Understanding these differences before surgery may help clinicians plan safer procedures, support more accurate radiological assessment, reduce the risk of complications, and provide valuable guidance for future studies in surgical planning and patient safety.

## 1. Introduction

The pterygomaxillary region is an anatomically complex area formed by the maxilla, palatine bone, and the pterygoid process of the sphenoid bone. This region contains the pterygopalatine fossa, which houses critical neurovascular structures, including the descending palatine canal. Preservation of these anatomical components during Le Fort I osteotomy is essential to prevent intraoperative bleeding, neurovascular injury, and other surgical complications [1].

Le Fort I osteotomy is a widely performed and reliable surgical technique for the correction of maxillofacial deformities [2]. In addition to providing aesthetic and functional improvement, it also allows access to anatomical regions such as the paranasal sinuses, nasopharyngeal area, and cranial base [3,4]. Due to its low complication rate, technical feasibility, and predictable outcomes, it is considered a standard approach in maxillofacial surgery [5]. However, the pterygomaxillary separation phase represents the most critical and complication-prone step of the procedure. During this stage, separation of the maxillary tuberosity from the pterygoid process under limited visibility may lead to complications such as hemorrhage, pterygoid lamina fracture, or nerve injury [6,7]. However, there is still a lack of clinically oriented morphometric evidence describing how anatomical variability in the pterygomaxillary region should be incorporated into osteotomy depth, separation direction, and safe disjunction limits. Most previous studies have reported descriptive measurements without directly linking these values to surgical decision-making. This gap limits the translation of anatomical knowledge into practical guidance for surgeons during the critical pterygomaxillary separation phase of Le Fort I osteotomy.

Variations in the morphology of the pterygomaxillary region such as the thickness of the pterygoid process, the lengths of the medial and lateral pterygoid laminae, and the width of the pterygomaxillary fissure can directly influence the safety of osteotomy procedures [8]. In particular, the course of the descending palatine artery is of critical importance for surgical planning; insufficient anatomical knowledge of this vessel may result in severe intraoperative hemorrhagic complications [9]. Therefore, detailed morphometric analysis of the pterygomaxillary region may contribute to defining safe surgical boundaries in Le Fort I osteotomies. By providing clinically interpretable CBCT-based morphometric measurements, the present study aims to identify anatomical thresholds that can assist surgeons in planning osteotomy depth, angulation, and separation strategy, thereby improving the safety of pterygomaxillary disjunction.

In recent years, cone-beam computed tomography (CBCT) has become widely used in the preoperative planning of maxillofacial surgery due to its low radiation dose, high spatial resolution, and ability to provide three-dimensional assessment [10]. In this study, the morphological characteristics of the pterygomaxillary region associated with Le Fort I osteotomy were evaluated using CBCT sections, and the effects of age, sex, and laterality on these parameters were analyzed.

## 2. Materials and Methods

Ethical approval for this study was obtained from the Clinical Research Ethics Committee of Dicle University Faculty of Dentistry (approval number: 2024-27, dated 27 November 2024). A total of 200 cone-beam computed tomography (CBCT) scans (100 males and 100 females) that met the study criteria were retrospectively analyzed. The mean age was 47.94 ± 14.69 years for males and 48.35 ± 13.64 years for females. Participants were categorized into three age groups: Group 1 (20–35 years), representing 20% (n = 40); Group 2 (36–50 years), representing 32% (n = 65); and Group 3 (51–80 years), representing 47.5% (n = 95). All CBCT scans were acquired at the Department of Oral and Maxillofacial Surgery, Dicle University Faculty of Dentistry, between December 2021 and September 2024.

A priori power analysis was performed using effect size estimates derived from previous studies, yielding an effect size of d = 0.52. Based on this value, a significance level of α = 0.05 and a power of 95% indicated that a minimum sample size of n = 196 would be sufficient. However, to enhance the statistical robustness of the study, the final sample size was increased to 200 participants.

The exclusion criteria were defined as the presence of bone defects or pathological lesions in the posterior maxillary region, a history of previous tumor or cyst surgery, a history of trauma or orthognathic surgery, and the presence of artifacts that adversely affected image quality. Consequently, CBCT data from 200 patients who met these criteria were included in the final analysis.

### 2.1. Imaging Protocol

CBCT data were obtained between December 2021 and September 2024 at the Department of Oral and Maxillofacial Surgery, Dicle University Faculty of Dentistry. All scans were acquired using an i-CAT^®^ Model 17–19 system (Imaging Sciences International, Hatfield, PA, USA). Imaging parameters were standardized as follows: 120 kVp, 5 mA, 8.9 s scan time, 13 × 16 cm field of view, 0.3 mm^3^ isotropic voxel size, and 0.3-mm slice thickness. All data were saved in Digital Imaging and Communications in Medicine (DICOM) format. Images were analyzed on an HP Envy 13-ah1xxx laptop with a 1920 × 1080-pixel display resolution and all measurements were performed using i-CAT Vision version 1.9.3.13.

In each scan, the patient’s head was positioned with the Frankfurt horizontal plane parallel to the floor and the midsagittal plane perpendicular to the floor. Head position was verified using a laser alignment system.

### 2.2. Standardization of Images

**Figure 1:**
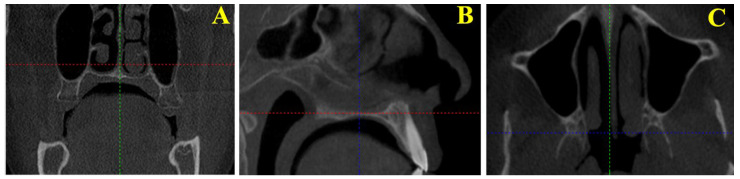
(**A**) Positioning of the sagittal reference line in the coronal plane by passing through the midpoint of the anterior nasal spine on cone-beam computed tomography (CBCT) images. (**B**) Identification of reference points in the sagittal plane for axial evaluation on CBCT images. (**C**) Selection of the axial slice that best visualizes the pterygomaxillary region between the predetermined reference points on CBCT images.

Based on previously described methods in the literature [7,8], certain modifications were implemented to enhance measurement repeatability. A sagittal reference line was aligned in the coronal plane by passing through the midpoint of the anterior nasal spine (Figure 1A). The Frankfurt horizontal plane was oriented parallel to the ground. Two reference points were identified at the level of the nasal floor (2 mm and 5.2 mm superior to it), and axial evaluations were performed between these reference points (Figure 1B).

This method allowed the standardization of the section planes in which the ptery-gomaxillary region could be most clearly visualized.

In the axial plane, images were aligned between the two reference points along the axial guide line, and the slice in which the pterygomaxillary region was most clearly visualized was identified. All measurements were then performed on this selected slice (Figure 1C).

### 2.3. Measured Morphometric Parameters

**Figure 2:**
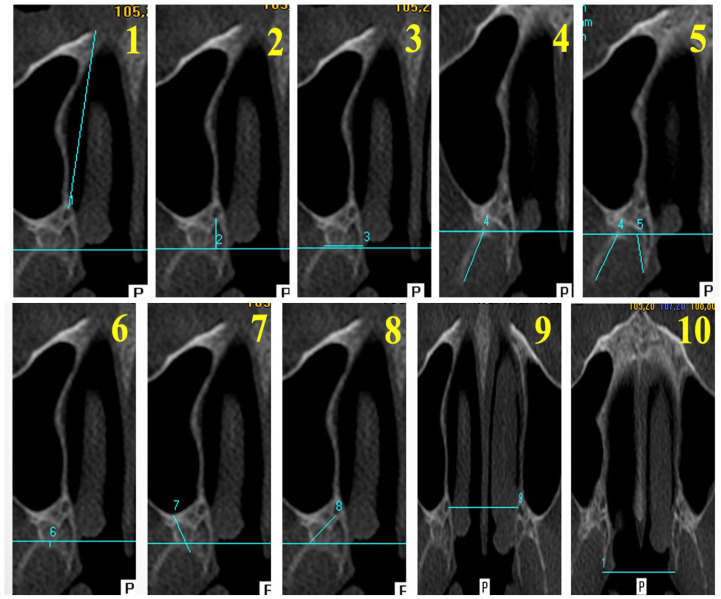
Axial CBCT measurements of the evaluated parameters. (**1**) AL—Anterior Length; (**2**) PL—Posterior Length; (**3**) PPW—Pterygoid Process Width; (**4**) PPT—Pterygoid Process Thickness; (**5**) LLL—Lateral Lamina Length; (**6**) MLL—Medial Lamina Length; (**7**) PMT—Pterygomaxillary Thickness; (**8**) DF–GPC—Distance between the Pterygomaxillary Fissure and the Greater Palatine Canal; (**9**) DGPC—Distance Between Greater Palatine Canals; (**10**) DML—Distance Between Medial Lamina Endpoints.

The measurement parameters evaluated in this study were selected based on previous morphometric investigations conducted using CBCT [7,8] (Figure 2). The definitions of the measurements are summarized below (Table 1).

All measurements were performed twice by the same researcher with a two-week interval. Intra-observer reliability was assessed using the Intraclass Correlation Coefficient (ICC), and ICC values were greater than 0.90 for all measurements.

### 2.4. Statistical Analysis

The normality of data distribution was assessed using the Shapiro–Wilk test. Parameters with normal distribution were presented as mean ± standard deviation, whereas non-normally distributed variables were expressed as median (min–max). Depending on data distribution, independent samples *t*-test, Mann–Whitney U test, ANOVA, or Kruskal–Wallis test was used for group comparisons. When statistically significant differences were detected, Bonferroni or Dunn–Bonferroni corrections were applied. In addition, multivariable linear regression analyses were performed to evaluate the independent associations of sex, age, and laterality with key morphometric measurements (AL, LLL, PMT, DGPC, and DML), in order to control for potential confounding effects. Statistical analyses were performed using SPSS version 25.0 (IBM Corp., Armonk, NY, USA), and a significance level of *p* < 0.05 was adopted.

## 3. Results

Values are presented as mean ± standard deviation for normally distributed variables and median (min–max) for non-normally distributed variables. *p*-values in the “SEX” rows indicate sex differences within the same side (female vs. male), whereas *p*-values in the “TOTAL” rows indicate side differences within the same sex (left vs. right). Statistical tests used for each comparison are indicated in the table footnotes, and Bonferroni (or Dunn–Bonferroni) corrections were applied where multiple comparisons were performed. Effect sizes have been added.

Measurement data obtained from CBCT scans of 200 individuals were analyzed according to age, sex, and laterality (Table 2). The overall data distribution was within normal limits, and the parameters demonstrating statistically significant differences are summarized below.

### 3.1. Anterior Length Measurements

No statistically significant difference was observed between sexes in anterior length measurements (*p* > 0.05). However, side comparison revealed significantly higher values on the left side compared to the right (*p* < 0.001). When evaluated across age groups, the third age group (51–80 years) exhibited a greater mean anterior length than the other groups. Minimum and maximum values measured on the left side were 33.6 mm and 42.2 mm, respectively, whereas on the right side they were 31.7 mm and 41.9 mm. These findings suggest that osteotomy depth should be planned with careful consideration of these boundary values during surgery.

In multivariable linear regression analysis, sex was not independently associated with AL after adjustment for age and laterality (β = −0.066, *p* = 0.164). In contrast, age (β = 0.264, *p* < 0.001) and laterality (β = 0.180, *p* < 0.001) remained significant independent predictors of AL.

### 3.2. Posterior Length Measurements

No statistically significant differences were observed in posterior length measurements according to sex, age, or laterality (*p* > 0.05). The values demonstrated a homogeneous distribution across all groups.

### 3.3. Measurements of Pterygoid Process Width

No statistically significant differences were found in pterygoid process width with respect to sex, age, or laterality (*p* > 0.05). The values demonstrated minimal variation among individuals.

### 3.4. Measurements of Lateral Pterygoid Lamina Length

No significant differences were detected in lateral pterygoid lamina length with respect to sex or laterality (*p* > 0.05). However, comparison across age groups revealed a statistically significant increase in lamina length in individuals within the third age group (*p* = 0.048) (Table 3). This finding suggests that age-related remodeling may occur in the lateral pterygoid lamina, potentially associated with changes in pterygoid muscle activity over time.

Values are presented as mean ± standard deviation. *p*-values indicate differences among age groups within the same side (Kruskal–Wallis test). When significant, pairwise post hoc comparisons were adjusted using the Dunn–Bonferroni correction.

In multivariable linear regression analysis, sex was not independently associated with LLL after adjustment for age and laterality (β = −0.002, *p* = 0.973). In contrast, age remained a significant independent predictor of LLL (β = 0.175, *p* < 0.001), whereas laterality was not significantly associated with LLL (β = 0.025, *p* = 0.612).

### 3.5. Measurements of Medial Pterygoid Lamina Length

No statistically significant differences were observed in medial pterygoid lamina length with respect to sex, age groups, or laterality (*p* > 0.05). Measurement values demonstrated similar ranges on both sides.

### 3.6. Measurements of Pterygoid Process Thickness

No statistically significant differences were detected in pterygoid process thickness with respect to sex, age, or laterality (*p* > 0.05). The measured values were consistent with mean values reported in the literature.

### 3.7. Measurements of Pterygomaxillary Region Thickness

When evaluated by sex, pterygomaxillary region thickness was significantly higher in females on both sides (*p* = 0.014). No significant differences were observed between sides or among age groups (*p* > 0.05). According to the literature, increased thickness of the pterygomaxillary region may reduce the risk of pterygoid lamina fracture; however, it may also increase the likelihood of crossing the greater palatine foramen line during osteotomy. Therefore, females may exhibit relatively greater resistance to lamina fractures, yet potentially a higher susceptibility to complications associated with the greater palatine foramen during surgery.

In multivariable linear regression analysis, sex remained independently associated with PMT after adjustment for age and laterality (β = 0.142, *p* = 0.004). In contrast, neither age (β = 0.022, *p* = 0.657) nor laterality (β = −0.035, *p* = 0.477) was significantly associated with PMT.

### 3.8. Measurements of the Distance Between the Pterygomaxillary Fissure and the Greater Palatine Canal

No statistically significant differences were observed in this parameter with respect to sex, age, or laterality (*p* > 0.05). Measurement values remained within a similar range across all groups.

### 3.9. Measurements of the Distance Between the Bilateral Greater Palatine Canals

No statistically significant differences were observed among age groups regarding the distance between the bilateral greater palatine canals (*p* > 0.05). However, males demonstrated significantly greater measurements compared to females (*p* < 0.001). This finding suggests that the maxillary base may be wider in males than in females.

In multivariable linear regression analysis, age was not independently associated with DGPC (β = −0.004, *p* = 0.954), whereas laterality remained a significant independent predictor (β = 0.297, *p* < 0.001). Sex could not be retained in the final model due to collinearity with laterality, which resulted in zero tolerance and prevented stable coefficient estimation.

### 3.10. Measurements of the Distance Between the Bilateral Medial Lamina Endpoints

No statistically significant differences were observed among age groups in the distance between the bilateral medial lamina endpoints (*p* > 0.05). However, males demonstrated significantly greater values compared to females (*p* < 0.001). This finding suggests that the pterygomaxillary complex may exhibit a wider morphological configuration in males.

In multivariable linear regression analysis, age was not independently associated with DML (β = 0.006, *p* = 0.935), whereas laterality remained a significant independent predictor (β = 0.278, *p* < 0.001). Sex could not be retained in the final model due to collinearity with laterality, resulting in zero tolerance.

In this study, significant differences were observed in several pterygomaxillary region parameters, including anterior length, lateral lamina length, pterygomaxillary region thickness, bilateral greater palatine canal distance, and the distance between the bilateral medial lamina endpoints. Sex, age, and laterality were found to play important roles in determining these anatomical variations.

## 4. Discussion

This study aimed to analyze the morphometric characteristics of the pterygomaxillary region using CBCT sections, as this anatomical area may contribute to complications during Le Fort I osteotomy. The findings demonstrated significant differences in pterygomaxillary parameters according to age, sex, and laterality. The data obtained hold clinical relevance, as they may assist in defining safe osteotomy boundaries and improving surgical planning.

Pterygomaxillary separation represents one of the most critical stages of Le Fort I osteotomy, and the majority of complications occur during this phase [9]. Serious adverse events such as pterygoid lamina fracture, maxillary artery injury, nerve damage, and excessive bleeding are largely associated with anatomical variations in this region [10,11,12]. Therefore, detailed knowledge of the anatomical characteristics of the pterygomaxillary complex directly influences surgical safety.

Ueki et al. [7] reported the distance between the piriform rim and the descending palatine artery as 39.1 ± 3.8 mm on the right side and 39.4 ± 4.0 mm on the left side. In a cadaver study, Li et al. [13] found this distance to be 38.4 mm in males and 34.6 mm in females. In the present study, the mean anterior length was measured as 38.11 ± 2.75 mm on the left side and 37.69 ± 2.85 mm on the right side, demonstrating consistency with the existing literature. Notably, the shortest recorded anterior distance was 31.74 mm, suggesting that the lateral nasal osteotomy during Le Fort I surgery should not extend beyond this limit.

Regarding the length of the lateral lamina, Ueki et al. [7] reported greater values on the left side, while Kanazawa et al. [14] reported a mean length of 11.4 ± 3.6 mm. In the present study, the mean lateral lamina length was 12.36 ± 4.70 mm on the left side and 12.14 ± 4.44 mm on the right side, with no sex-related differences. However, a tendency for increased length with advancing age was observed. This increase may be associated with age-related remodeling of the lateral pterygoid lamina, potentially influenced by prolonged functional activity of the lateral pterygoid muscle [15].

Regarding pterygomaxillary region thickness (PMT), Hwang et al. [16] reported mean values of 7.70 ± 3.67 mm in the successful separation group and 4.70 ± 2.59 mm in the fracture group. Kanazawa et al. [14] measured an average thickness of 2.6 ± 1.7 mm and suggested that decreased thickness increases the risk of lamina fracture. In the present study, the mean PMT was 4.42 ± 3.36 mm on the right side and 4.20 ± 2.77 mm on the left side, with values significantly higher in females (*p* < 0.01). These findings support the assumption that lamina fractures may occur more frequently in males, who present with a thinner pterygomaxillary region.

Lee et al. [8] reported the bilateral greater palatine canal distance as 34.4 ± 3.3 mm in the cleft group and 34.3 ± 2.5 mm in the control group. In a cadaver study, Dave et al. [17] found this distance to be 33.3 mm in males and 32.6 mm in females. In the present study, the mean distance was 31.32 ± 3.04 mm in males and 29.54 ± 2.68 mm in females, with the difference being statistically significant (*p* < 0.01). These findings support the notion that males possess a wider maxillary base structure compared to females.

Similarly, the distance between the bilateral medial lamina endpoints was measured as 30.84 ± 2.79 mm in males and 29.21 ± 2.88 mm in females, with this difference being statistically significant (*p* < 0.01). This finding indicates that the pterygomaxillary complex generally exhibits a wider morphological configuration in males. Overall, the results are consistent with previously reported measurements in the literature, although sex- and age-related variations were observed in certain parameters.

Because pterygomaxillary separation during Le Fort I osteotomy is typically performed under limited or blind visualization, morphological variations in this region directly influence the risk of complications. In particular, anatomical differences in the course of the descending palatine artery and the pterygoid venous plexus may increase the likelihood of intraoperative bleeding or vascular injury.

The findings of this study indicate that the pterygomaxillary region tends to be thicker in females, whereas males generally exhibit a wider maxillary base. This difference highlights the importance of adjusting the depth and direction of the osteotomy line according to sex during surgical planning. Additionally, the age-related increase in lateral lamina length may reduce the risk of pterygoid plate fracture; however, it may also require greater force to complete the osteotomy.

This study has several limitations. First, it was designed as a retrospective analysis; therefore, prospective and multicenter studies may provide stronger evidence. Second, the sample was obtained from a specific region of Türkiye, and anatomical variations may differ across ethnic populations. Third, measurements were performed solely on axial sections; incorporating coronal and sagittal planes and conducting three-dimensional analyses could be beneficial in future research. Finally, functional and clinical correlations, such as postoperative complication rates, were beyond the scope of the present study.

From a clinical perspective, detailed CBCT evaluation of the pterygomaxillary region prior to Le Fort I osteotomy enables the identification of individual anatomical variations and enhances surgical safety. Sex- and age-related morphometric differences should be considered in surgical planning, and the depth and direction of osteotomy cuts should be adjusted accordingly. Future studies incorporating three-dimensional modeling and virtual surgical simulation across diverse populations may further clarify the relationship between pterygomaxillary anatomical variations and surgical risk.

## 5. Conclusions

This study demonstrated that the morphometric characteristics of the pterygomaxillary region vary significantly according to age, sex, and laterality. Given that most complications during Le Fort I osteotomy occur at the pterygomaxillary separation stage, consideration of these anatomical variations during preoperative assessment is of great importance.

- The anterior length was significantly greater on the left side, indicating that this factor should be considered when planning the depth of the osteotomy.
- Increased pterygomaxillary region thickness in females may serve as a potential protective factor against lamina fractures; however, caution is warranted regarding osteotomy lines that may pass near the greater palatine foramen.
- The greater inter-canalis palatinus majus distance and medial lamina endpoint distance observed in males suggest a generally wider morphological configuration of the pterygomaxillary complex in this population.
- The age-related increase in lateral lamina length supports the possibility of functional remodeling of the pterygoid plate morphology over time.

Based on these findings, individualized CBCT-based morphometric evaluation of the pterygomaxillary region prior to Le Fort I osteotomy is recommended to reduce intraoperative complication risk and enhance surgical safety.

Planning approaches that consider sex- and age-related morphological variability of the pterygomaxillary complex are expected to reduce complication rates and improve postoperative functional and aesthetic outcomes in the future.

## Figures and Tables

**Table 1:** Descriptions of the measured parameters.

ABBREVİATİON	PARAMETER NAME	DEFİNİTİON
Anterior Length (AL)	Anterior Length Measurements	Distance from the descending palatine artery to the piriform rim
Posterior Length (PL)	Posterior Length Measurements	Distance from the posterior point of the greater palatine canal to the deepest concavity of the pterygomaxillary fissure
Pterygoid Process Width (PPW)	Measurements of Pterygoid Process Width	Minimum width of the pterygoid process along the pterygomaxillary fissure line
Lateral Lamina Length (LLL)	Measurements of Lateral Pterygoid Lamina Length	Distance from the deepest concavity of the pterygomaxillary fissure to the posterior-most point of the lateral lamina
Medial Lamina Length (MLL)	Measurements of Medial Pterygoid Lamina Length	Distance from the most medial point of the fissure to the posterior end of the medial lamina
Pterygoid Process Thickness (PPT)	Measurements of Pterygoid Process Thickness	Distance from the deepest point of the pterygoid fossa to the pterygomaxillary fissure
Pterygomaxillary Region Thickness (PMT)	Measurements of Pterygomaxillary Region Thickness	The minimum distance between the posterior wall of the maxillary sinüs and the deepest point of the pterygoid fossa
**Distance Fissure to Greater Palatine Canal** (DF–GPC)	Measurements of the Distance Between the Pterygomaxillary Fissure and the Greater Palatine Canal	Distance from the most concave point of the lateral portion of the pterygomaxillary fissure to the greater palatine canal
Distance Between Greater Palatine Canals (DGPC)	Measurements of the Distance Between the Bilateral Greater Palatine Canals	Distance between the most medial points of the right and left greater palatine canals
Distance Between Medial Lamina Endpoints (DML)	Measurements of the Distance Between the Bilateral Medial Lamina Endpoints	Distance between the medial lamina endpoints of the right and left pterygoid processes

The following abbreviations were used for morphometric parameters: AL (Anterior Length), PL (Posterior Length), PPW (Pterygoid Process Width), PPT (Pterygoid Process Thickness), LLL (Lateral Lamina Length), MLL (Medial Lamina Length), PMT (Pterygomaxillary Thickness), DF–GPC (Distance between the Pterygomaxillary Fissure and the Greater Palatine Canal), DGPC (Distance Between Greater Palatine Canals), and DML (Distance Between Medial Lamina Endpoints).

**Table 2:** Comparison of morphometric parameters by sex and side.

PARAMETERS	SIDE	SEX	N	MEAN	STANDARD DEVIATION	η^2^	*p*
AL	LEFT	FEMALE	100	38.16	1.97	0.0037	0.391 ^c^
		MALE	100	38.45	1.55		
		TOTAL	200	38.31	1.78	0.0320	<0.001 ^a^
	RIGHT	FEMALE	100	37.57	2.11	0.002	0.529 ^a^
		MALE	100	37.73	1.50		
		TOTAL	200	37.65	1.83	0.0320	<0.001 ^a^
PL	LEFT	FEMALE	100	2.10	1.53	0.0003	0.814 ^c^
		MALE	100	2.14	1.75		
		TOTAL	200	2.12	1.64	0	0.903 ^c^
	RIGHT	FEMALE	100	2.12	1.81	0	0.991 ^c^
		MALE	100	2.14	1.79		
		TOTAL	200	2.13	1.79	0	0.903 ^c^
PPW	LEFT	FEMALE	100	7.54	2.25	0.0056	0.292 ^c^
		MALE	100	7.85	2.30		
		TOTAL	200	7.69	2.27	0.0049	0.162 ^c^
	RIGHT	FEMALE	100	7.89	2.59	0.0035	0.403 ^c^
		MALE	100	8.21	2.54		
		TOTAL	200	8.05	2.57	0.0049	0.162 ^c^
LLL	LEFT	FEMALE	100	12.37	4.88	0	0.972 ^c^
		MALE	100	13.36	4.54		
		TOTAL	200	12.36	4.70	0.0009	0.561 ^c^
	RIGHT	FEMALE	100	12.14	4.48	0	0.901 ^c^
		MALE	100	12.14	4.43		
		TOTAL	200	12.14	4.44	0.0009	0.561 ^c^
MLL	LEFT	FEMALE	100	7.44	3.31	0	0.992 ^c^
		MALE	100	7.41	3.52		
		TOTAL	200	7.42	3.40	0.0005	0.655 ^c^
	RIGHT	FEMALE	100	7.35	2.66	0.0022	0.509 ^c^
		MALE	100	7.11	2.82		
		TOTAL	200	7.23	2.73	0.0005	0.655 ^c^
PPT	LEFT	FEMALE	100	1.18	2.51	0.0025	0.477 ^c^
		MALE	100	0.95	2.53		
		TOTAL	200	1.06	2.52	0.0013	0.471 ^c^
	RIGHT	FEMALE	100	1.40	2.06	0.0158	0.076 ^c^
		MALE	100	0.92	2.33		
		TOTAL	200	1.16	2.20	0.0013	0.471 ^c^
PMT	LEFT	FEMALE	100	4.63	2.81	0.0328	0.010 ^c^
		MALE	100	3.78	2.68		
		TOTAL	200	4.20	2.77	0.0003	0.754 ^c^
	RIGHT	FEMALE	100	4.88	3.47	0.0305	0.014 ^c^
		MALE	100	3.97	3.21		
		TOTAL	200	4.42	3.36	0.0003	0.754 ^c^
DF–GPC	LEFT	FEMALE	100	5.51	2.92	0	0.976 ^c^
		MALE	100	5.55	2.50		
		TOTAL	200	5.53	2.71	0.0043	0.187 ^c^
	RIGHT	FEMALE	100	5.67	2.99	0.0087	0.187 ^c^
		MALE	100	6.22	2.98		
		TOTAL	200	5.95	2.99	0.0043	0.187 ^c^
DGPC		FEMALE	100	29.54	2.68	0.0796	<0.001 ^c^
		MALE	100	31.32	3.04		
DML		FEMALE	100	29.21	2.88	0.0783	<0.001 ^c^
		MALE	100	30.84	2.79		

a: Independent samples *t*-test. c: Mann–Whitney U test. Post hoc: Bonferroni/Dunn–Bonferroni correction applied where appropriate.

**Table 3:** Comparison of lateral lamina length (LLL) measurements across age groups.

	SIDE	N	AGE GROUP	MEAN	STANDARD DEVIATION	η^2^	*p*
LLL	LEFT	200	20–35 (n = 40	11.56	3.52	0.021	0.043 ^d^
			36–50 (n = 65)	11.97	5.72		
			51–80 (n = 95)	12.99	4.30		
	RIGHT	200	20–35 (n = 40	10.93	3.66	0.021	0.048 ^d^
			36–50 (n = 65)	11.76	4.37		
			51–80 (n = 95)	12.91	4.69		

d: Kruskal–Wallis test. Post-hoc: Bonferroni/Dunn–Bonferroni correction applied where appropriate.

## Data Availability

The original contributions presented in this study are included in the article. Further inquiries can be directed to the corresponding author.

## Abbreviations

The following abbreviations are used in this manuscript:

CBCT	Cone Beam Computed Tomography
DICOM	Digital Imaging and Communications in Medicine
AL	Anterior Length
PL	Posterior Length
PPW	Pterygoid Process Width
PPT	Pterygoid Process Thickness
LLL	Lateral Lamina Length
MLL	Medial Lamina Length
PMT	Pterygomaxillary Thickness
DF-GPC	Distance between the Pterygomaxillary Fissure and the Greater Palatine Canal
DGPC	Distance Between Greater Palatine Canals
DML	Distance Between Medial Lamina Endpoints
ICC	Intraclass Correlation Coefficient
